# Modified Hematopoietic Stem Cell-Derived Dendritic Cell Therapy Retained Tumor-Inhibitory Function and Led to Regression of Primary and Metastatic Pancreatic Tumors in Humanized Mouse Models

**DOI:** 10.3390/vaccines13111131

**Published:** 2025-11-02

**Authors:** Jose D. Gonzalez, Saleemulla Mahammad, Senay Beraki, Ariel Rodriguez-Frandsen, Neha Sheik, Elango Kathirvel, Francois Binette, David Weinstein, Anahid Jewett, Lu Chen

**Affiliations:** 1 Renovaro Biosciences, 2080 Century Park East, Suite 906, Los Angeles, CA 90067, USA; 2 Division of Oral Biology and Medicine, The Jane and Jerry Weintraub Center for Reconstructive Biotechnology, Los Angeles School of Dentistry, University of California, 10833 Le Conte Ave, Los Angeles, CA 90095, USA; 3 The Jonsson Comprehensive Cancer Center, Los Angeles School of Dentistry and Medicine, University of California, Los Angeles, CA 90095, USA

**Keywords:** hematopoietic stem cells, humanized mouse models, vector optimization, DC therapy, CD40L, CD93, CXCL13, pancreatic cancer, in vivo live imaging, allogeneic engineered DCs

## Abstract

**Background/Objectives**: Dendritic cell (DC)-based immunotherapies offer a promising strategy for cancer treatment but are limited by inefficient activation of cytotoxic T cells and, in turn, the host immune system. This report demonstrated that CD34^+^ hematopoietic stem cell (HSC)-derived allogeneic DCs engineered by an optimized lentiviral vector (LVV) expressing CD93, CD40-ligand (CD40L), and Chemokine (C-X-C motif) ligand-13 (CXCL13) significantly enhanced the host immune system, activated tumor-specific cytotoxic T cells, and led to complete regression of both primary and metastatic pancreatic tumors in humanized mouse models. This LVV shows comparable pre-clinical efficacy compared to the first-generation vector, in addition to being compliant for clinical use, which allows further pre-clinical development towards the human trials. **Methods**: This 2nd generation (Gen) LVV incorporates codon-optimized transgenes (CD40L, CD93, and CXCL13) with rearranged sequence to enhance expression, driven by a strong EF1α promoter. CD34^+^ HSCs were transduced with this modified 2nd Gen LVV and differentiated to Engineered DCs. Therapeutic efficacy of the DC therapy with the modified vector was tested on humanized mouse models of pancreatic tumors. This was accomplished by establishing an early-stage disease model (using MIA PaCa-2 (MP2)-tumors) and late-stage metastatic disease model of the pancreatic tumors to mimic the clinical setting using luciferase-expressing MP2-(Luc)-pancreatic tumor-bearing humanized mice. **Results**: The modified lentiviral construct had 6-fold greater expression of CD40L, 2% less toxicity, 4.5-fold greater CD40L, and 2.2-fold greater CXCL13 secretion than its predecessor. In vitro, Engineered DCs induced robust T cell proliferation in up to 20% of T cells, up to 4-fold greater interferon-gamma (IFN-γ) secretion than controls, and showcased antigen-specific cytotoxicity by CD8^+^ T cells. In vivo, two intradermal doses of the 2nd Gen DCs led to complete regression of primary pancreatic tumors and metastases. Treated mice exhibited prolonged survival, indicating the induction of durable anti-tumor immunity. **Conclusions**: Vector optimization retained the efficacy of DC-based therapy, achieving curative responses in pancreatic tumor models. These findings support the clinical development of this 2nd Gen DC immunotherapy for pancreatic and potentially other tumors.

## 1. Introduction

Pancreatic cancer (PC), with its high mortality rate and dismal prognosis, is the third leading cause of cancer-related death in the U.S. Key risk factors include smoking, obesity, and diabetes. While traditional treatments like surgery and chemotherapy are available, their effectiveness is limited due to late-stage diagnosis. Although immunotherapy has revolutionized the treatment of many cancers, its effectiveness in alleviating the burden of PC has been met with many challenges. Several factors complicate the immune system’s ability to recognize and attack cancer cells, including the dense tumor microenvironment, which inhibits the infiltration of immune cells and acts as a physical barrier limiting the presence of immunosuppressive cells as well as the tumor’s low antigenicity [1,2]. Arguably the most potent antigen-presenting cell (APC) type that bridges innate and adaptive immunity, the Dendritic cell (DC) is capable of priming naïve T cells and initiating effective adaptive immune responses against infection and cancer [3]. Over the last two decades, DC-based therapies have been explored extensively as an immunotherapeutic treatment option for a wide variety of cancers, most notably melanoma, glioblastoma, prostate cancer, and PC [4,5]. Clinical studies have shown that ex vivo-generated DCs loaded with tumor antigens can induce tumor-specific cytotoxic T lymphocyte (CTL) responses and, in some cases, durable clinical remissions [6,7]. For instance, the approval of Sipuleucel-T (Provenge^®^) for metastatic prostate cancer established the feasibility of autologous DC vaccines as a therapeutic approach [8]. However, autologous DC therapies face or are limited by multiple challenges, particularly when it comes to the treatment of solid tumors. These include the time and labor-intensive manufacturing process, variability of patients, limited availability of circulating monocytes as a starting material, and insufficient immunogenicity in the highly suppressive tumor microenvironment (TME) [9]. Solid tumors, such as PC, present additional challenges due to a dense desmoplastic stroma, low effector T-cell infiltration, and the presence of immunosuppressive cells that inhibit anti-tumor immune responses [10,11]. Recent studies were conducted with efforts to improve DC therapies that were focused on enhancing antigen loading, co-stimulation, cytokine secretion, and migratory capacity, often through genetic engineering approaches.

In our recently published study [12], we have described a novel CD34^+^ hematopoietic stem cell (HSC)-derived allogeneic DC therapy that was lentivirally engineered to express three key immunomodulatory molecules: CD93, CD40-ligand (CD40L), and Chemokine (C-X-C motif) ligand-13 (CXCL13) via lentiviral transduction. CD93, a C-type lectin-like protein, promotes immune cell infiltration and phagocytosis, critical for overcoming the immunosuppressive tumor microenvironment [13]. CD40L, expressed on activated T cells and DCs, amplifies T cell priming through bidirectional CD40-CD40L interactions [14]. CXCL13, a B-cell chemoattractant, enhances lymphoid tissue recruitment, potentially boosting humoral and T-helper responses [15]. Engineered DCs expressing these three factors exhibited enhanced capacity for antigen presentation, T-cell activation, and recruitment of immune effector cells. In humanized mouse models of orthotopic PC, treatment with these Engineered DCs led to significant tumor reduction, increased tumor-infiltrating lymphocytes (CD4^+^ and CD8^+^ T cells), and improved survival. Lentiviral vector (LVV) was used to deliver the three target genes. LVV could efficiently deliver and permanently integrate genetic material into the genome of the cells, lowering stable long-term expression of the therapeutic genes [16]. It has been used clinically in the chimeric receptor antigen T-cell (CAR-T) therapies. The major risk of LVV is insertional mutagenesis from random integration into the host genome. However, the current clinical experience of the LVV showed that insertional oncogenesis was rare. Allogeneic DCs edited by the LVV impose even lower risk, as the DCs would be quickly removed by the host immune responses.

In this follow-up study, we optimized the LVV design to enhance the three target gene expressions and to incorporate a clinical compliant design for future potential clinical development. We rearranged transgenes in the order CD40L-CD93-CXCL13 and utilized a strong EF1α promoter to drive robust expression of these three transgenes. We hypothesized that these optimizations would improve DC maturation, cytokine production, and T-cell priming and translate into superior anti-tumor responses. Additionally, the LVV was modified for potential future clinical applications with the resistance gene changed from ampicillin to kanamycin and the expression regulatory element changed from wPRE to oPRE. While comparable in terms of transgene expression, oPRE is often preferred due to its smaller size and safety profile, which are essential for regulatory compliance [17,18]. Furthermore, in the optimized LVV, the antibiotic resistance was changed to Kanamycin to minimize the risk of β-lactam–induced hypersensitivity reactions in patients and to reduce the potential spread of β-lactamase resistance genes to environmental microbes, which is considered more stable and broadly accepted for use in vectors intended for biological production [19,20]. The allogeneic DCs transduced with the optimized 2nd generation (Gen) LVV were tested both in vitro and in vivo to ensure they have comparable or better performance than the 1st Gen product.

Here, we report preliminary observational data that this 2nd Gen Engineered DC therapy not only induces robust immune activation but also achieves complete regression of both primary pancreatic tumors and metastatic lesions in vivo in a head-to-head comparison with our 1st Gen Engineered DC therapy. Furthermore, we optimized our pancreatic tumor model by using Luciferase-expressing MIA PaCa-2 (MP2-Luc) cells, enabling visualization of tumors in live mice and analysis of primary and metastatic lesions. This disease model with MP2-Luc cells mimicked a late-stage pancreatic tumor model with metastatic lesions and poor survival. Treatment of these MP2-Luc tumor-bearing humanized mice revealed that optimized Engineered DCs not only reduce tumor burden but also achieve complete regression of both primary pancreatic tumors and metastatic lesions. Although these results are observational preliminary findings, we believe this tumor-killing response warrants further investigation into the specific immune activation mechanics driving this platform and showcases potential for the treatment of PC and other solid tumor types.

## 2. Materials and Methods

### 2.1. Cells and Reagents

Standard conditions were used to culture all cells at 37 °C and 5% CO_2_ in a humidified incubator. Prior to use in experiments, all cells were evaluated for mycoplasma contamination. Dulbecco’s Modified Essential Medium (DMEM) (Gibco, Billings, MA, USA) enriched with fetal bovine serum (FBS) (ThermoFisher, Waltham, MA, USA, Cat# A5669701) to a final concentration of 10% was used to culture pancreas carcinoma MP2 cancer cells (American Type Culture Collection (ATCC), Manassas, VA, USA, Cat# CRL-1420). MP2-Luc cells (GenTarget INC, San Diego, CA, USA, Cat# SC079-LG) were cultured as per the provider’s recommendations using DMEM supplemented with 10% heat-inactivated FBS + 1% Penicillin–Streptomycin (Gibco, Cat #1514012). RPMI 1610 Medium (Gibco, Cat# 11875093) supplemented with FBS to a final concentration of 10% was used to culture Jurkat T-cells [Clone E6-1, TIB-152] (ATCC), Peripheral Blood Mononucleated Cells (PBMCs) (isolated from mice), splenocytes (isolated from mice), and T cells (ATCC [PCS-800-016]).

### 2.2. Lentiviral Vectors and Engineered DC Generation

The 1st Gen LVV was constructed at VectorBuilder (Chicago, IL, USA) and included transgenes separated by P2A and T2A linkers in the order of CD93, CD40L, and CXCL13, as described in our previous publication [12]. The transgenes were expressed under EFS promoter, and the vector contained an ampicillin resistance gene. Three major modifications were made in the development of the 2nd Gen LVV. First, promoter EF1α was selected to enhance transgene expression. Second, while the same three genes were used, their order was changed to CD40L–CD93–CXCL13. Third, the Ampicillin resistance gene was replaced by the Kanamycin gene for the antibiotic selection, enabling regulatory, safety, and practical considerations relevant to clinical applications and gene therapy. In addition, woodchuck hepatitis virus posttranscriptional regulatory element (wPRE) was replaced by an optimized and shorter version, oPRE [21].

Human CD34+ HSCs were obtained from healthy donors under IRB-approved protocols and transduced with the optimized 2nd Gen LVV. All transductions using LVV were performed as described before in our previous publication [12], and said engineered cells were further expanded for 120 h following the 24 h incubation with LVVs. The cells were further differentiated into monocytes and then DCs as per the previously published protocol [12]. Experimental parameters, including culture conditions and incubation times, were consistent with those described previously [12]. Quality control measures, including flow cytometry and quantitative PCR (qPCR), were adapted and used to characterize cell phenotype and transduction efficiency. Controls, such as untransduced (UTD) cells and cells transduced with a lentiviral stuffer-sequence, were included to confirm specificity. Lysate production from MP2 cells or tumor tissue samples, which consists of 10 freeze-dry cycles and sonication of cells, and pulsing of mature DCs (mDCs) with the lysate, was performed as described [12]. The antigen education or pusling of the cells consists of incubating the DCs with the whole cell tumor lysate for 18 h at 18 µg per 10^6^ cells. We classify the resulting DCs as Engineered DCs.

### 2.3. Antibodies and Flow Cytometry

Flow cytometry was utilized to perform immunophenotyping of cells. REA monoclonal antibodies (mAbs) (Miltenyi Biotec, Bergisch Gladbach, Germany), including allophycocyanin-Cy7 (APC-Cy7)-conjugated anti-CD40L (clone REA238), fluorescein isothiocyanate (FITC)- and VioBlue (VB)-conjugated anti-CD3 (clone REA613), FITC-conjugated anti-CD93 (clone REA1111), and VB-conjugated anti-CD34 (clone REA1164) were utilized to perform cell surface staining as recommended by manufacturers. Following the manufacturer’s recommendations, human anti-CD3/CD28 mAbs for T cells were used and obtained from STEMCELL Technologies. Propidium iodide or 7AAD (Miltenyi Biotec) was used to evaluate cell viability. MACSQuant Analyzer 10 (Miltenyi) or Beckman Coulter Epics XL Cytometer (Brea, CA, USA) was used to perform flow cytometry, and this data was analyzed using FlowJo software (v10.10.0).

### 2.4. Enzyme-Linked Immunosorbent Assay (ELISA)

All ELISAs were conducted as outlined in prior reports [12]. CXCL13/BLC/BCA-1 ELISA Kit (ThermoFisher, Cat# EHCXCL13) and CD40 Ligand/TNFSF5 Quantikine ELISA Kit (R&D Systems, Minneapolis, MN, USA, Cat# DCDL40) were used following manufacturers’ recommendations to quantify secreted CXCL13 and CD40L collected from engineered cell supernatants. Human interferon-gamma (IFN-γ) ELISA Kit (ThermoFisher, Cat# KHC4021) was used to quantify IFN-γ secretion in response to activation caused by treatment with Engineered DCs in isolated ex vivo experiments. ELISA plates were analyzed using Varioskan Lux plate reader (ThermoFisher).

### 2.5. Mixed Lymphocyte Reaction (MLR) and T-Cell Proliferation

A one-way MLR assay was employed to measure T-cell proliferation against tumor-antigen-pulsed engineered DCs carrying the 2nd Gen vector. This protocol was adapted from Mangelinck et al. [22] with some modifications. Cryopreserved T cells were thawed at 37 °C, transferred dropwise into T-cell medium (3% human AB (ThermoFisher Cat# J66674.03), 97% TexMACS medium (Miltenyi Biotec, Cat# 130-097-196). These cells were centrifuged at 500× *g*, resuspended in 1× DPBS, and stained with 10 μM CellTrace Violet (ThermoFisher Cat# C34557). The Engineered DCs were used as stimulatory cells and co-cultured with CellTrace Violet-labeled T cells at different ratios, including 1:1, 1:2, 1:4, and 1:8 (DC: T cell) in 96-well plates. The cultures were incubated at 37 °C with 5% CO_2_ for 94 h. T-cell activation was assessed by flow cytometry by evaluating the CellTrace Violet dye (dilution) as a measurement of cell proliferation. Controls for this experiment included unstimulated T cells, no antigen-pulsed DC, and CD34^+^ HSCs. Analyzed using NovoExpress Software 1.6.3.

### 2.6. Enzyme-Linked Immunospot (ELISpot)

IFN-γ ELISpot assay was performed using Human IFN-γ ELISpot Plus kit (Mabtech, Nacka Strand, Sweden, Cat#3420-4APW-2) according to the manufacturer’s protocol. The assay was performed on a 96-well polyvinylidene fluoride-backed microtiter plate coated with capture antibody specific to IFN-γ in PBS. After incubating overnight at 4 °C, the plates were washed with PBS to remove unbound antibody and blocked with RPMI-1640 (supplemented with 10% FBS). After decanting the supernatant in the plates, the T cells and Engineered DCs were added to the wells and incubated at 37 °C in a humidified incubator for 48 h. Post-stimulation, the plates were washed five times with PBS, and 1 µg/mL of biotinylated anti-IFN-γ detection antibody was added. Plates were incubated at room temperature for 2 h and washed five times with PBS afterwards. Streptavidin-ALP diluted to 1:1000 in PBS was added to the plate and incubated at room temperature for 1 hr. After five additional washes, substrate solution was added to each well and incubated for 20 min at room temperature in the dark until spots developed. The plates were washed with distilled water to stop color development. Finally, the wells were air-dried at room temperature, spot-forming cells (SFCs) were quantified using Mabtech ASTOR ELISpot reader, and analysis was performed using Mabtech Apex™ RAWspot™ software v1.

### 2.7. Orthotopic Tumor Implantations and Treatment with DCs in Hu-BLT Mice

The University of California, Los Angeles (UCLA) animal facility provided the Humanized-Bone–Liver–Thymus (Hu-BLT) mice. Orthotopic implantation of pancreatic tumors using MP2 cells was performed according to previously described methods [12,23]. 12 Mice (3 per experimental group) were allowed to acclimate to the environment 168 h prior to the start of the experiment. Sample size was chosen to minimize costs and for statistical significance. Mice were individually identified and housed in groups of 2-to-5 in ventilated cages (type II, 16 × 19 × 35 cm, floor area = 500 cm^2^) under the following controlled conditions such room temperature (22 ± 2 °C), hygrometry (55 ± 10%), Photoperiod (12:12 h light–dark cycle 7 am:7 pm). Mouse handling, care, and treatment plan was performed as described in this study [12]. Water and food were available ad libitum. Mice were randomly divided into four groups: those receiving 2 injections of 10^6^ tumor-antigen-pulsed 1st Gen Engineered DCs, those receiving 2 injections of 10^6^ tumor-antigen-pulsed 2nd Gen Engineered DCs, or those receiving 2 injections of 10^6^ tumor-antigen-pulsed UTD DCs via intradermal (i.d.) injection adjacent to the surgical wound near the lymph node, and one group left untreated as a control. Mice were monitored for 5 weeks post-treatment for health and tumor responses. At the study endpoint, blood and tissue samples were collected and analyzed, and metastases and ascites were evaluated as described herein [24]. All animal housing and experimental procedures were reviewed and approved by the UCLA Animal Research Committee (ARC) and conducted in full compliance with state, federal, and institutional animal welfare guidelines. Ethic Committee Name: UCLA Animal Research Committee. Approval Code: ARC-2012-101. Approval Date: 18 December 2013.

### 2.8. PBMC and Splenocyte Isolation and Ex Vivo Activation Assay

PBMCs and splenocytes were isolated using the ficoll-hypaque method as described here [25,26]. The purity and viability of the cells were assessed using flow cytometry and trypan blue exclusion, respectively. Cells from each group were cultured for 120 h in the presence of anti-CD3/28. Supernatants were collected on day (D) 5 by centrifugation at 575× *g* for 5 min followed by aspirating the supernatant from the pelleted cells, and ELISA was conducted to analyze IFN-γ secretion. IFN-γ levels (pg/mL) are presented as mean ± standard deviation (SD).

### 2.9. Orthotopic Implantation of Luciferase-Expressing MP2 Tumors in CD34^+^ NCG Humanized Mice, Treatment Regimen with DCs, and In Vivo Live Imaging of Tumor Progression

Experiments were carried out with female NOD-Prkdc^em26Cd52^Il2rg^em26Cd22^/GptCrl immunodeficient (NCG) mice from Charles River Laboratories (Wilmington, MA, USA). Mice were humanized by using CD34^+^ HSCs from two donors that were isolated from human cord blood samples (TransCure, Farmers Branch, TX, USA). Only mice with an above 25% humanization rate (hCD45/totalCD45) were used for the study. The HLA-A2 status of the donors was recorded. Orthotopic injection of luciferase-encoded PC cells was performed as described here [27]. Six Mice (three per experimental group) were allowed to acclimate to the environment 168 h prior to the start of the experiment. Mice were individually identified and housed by groups of 2-to-5 in ventilated cages (type II, 16 × 19 × 35 cm, floor area = 500 cm^2^) under the following controlled conditions such as room temperature (22 ± 2 °C), hygrometry (55 ± 10%), photoperiod (12:12 h light–dark cycle 7 am:7 pm), and water and food available ad libitum.

Dosing of 2nd Gen Engineered DC treatment was performed post intra-pancreatic tumor implantations on D29 and repeated on D36; a total of two doses/group. Treatment consisted of 100 μL (2 × 50 μL) intradermal injections at a site close to lymph nodes on the right flank of the mouse. Mice were treated with 10^6^ cells for each treatment. Progression of luciferase-expressing tumors was monitored by in vivo imaging using a Vilber Newton 7.0. Prior to each imaging timepoint, mice were anesthetized with isoflurane and injected intraperitoneally with 150 mg/kg luciferin. All procedures described in this study have been reviewed and approved by the Local Ethics Committee for Animal Experimentation (CELEAG) under compliance with the Institutional Animal Care and Use Committee (IACUC). The Ethic Committee Name: CELEAG-TCS. Approval Code: A7418324. Approval Date: 9 December 2021. No data regarding animals was excluded.

### 2.10. Statistical Analyses

Statistical analysis of the results was performed by using Student’s t test (two groups), ANOVA (analysis of variance), and/or Tukey’s test (three or more groups) (****—*p* value < 0.0001, ***—*p* value < 0.001, **—*p* value < 0.001–0.01, and *—*p* value < 0.01–0.05). The Mann–Whitney U test was performed for non-normal data for the comparison of two groups. For comparing multiple groups, bonferroni correction was utilized and adjusted α = 0.05/2 = 0.025. Statistical analysis was performed using GraphPad Prism-10.3.0.507 software.

## 3. Results

### 3.1. LVV Optimization

**Figure 1:**
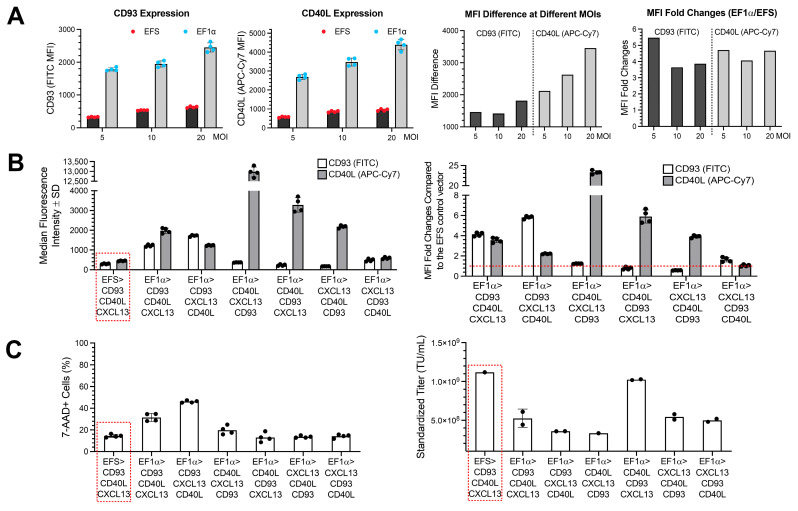

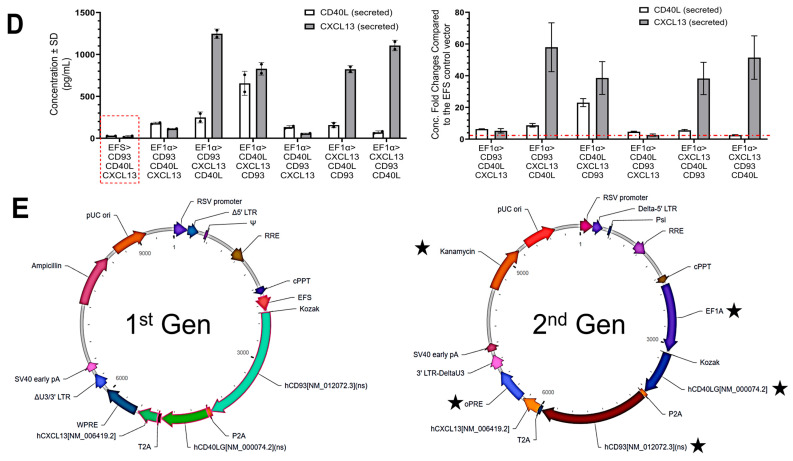
LVV Optimization. (**A**) Flow cytometry results of feasibility study testing the effect of varying MOIs (5, 10, and 20) and promoter strength (EFS vs. EF1α) on MFI of CD93 and CD40L expression illustrated in raw MFI, difference in MFI between the EFS and EF1α viruses at indicated MOI, and fold change, *n* = 4. EFS virus results are identified as blue points whereas EF1α virus results are identified as red points. (**B**) MFI results of CD93 and CD40L with EFS (EFS_CD93_CD40L_CXCL13) and six EF1α viruses (all possible combinations of transgene orientations) illustrated as MFI mean ± SD on the left and MFI fold changes relative to the EFS control virus on the right graph, *n* = 4, MOI = 5. EFS virus is identified by the red dashed box on left graph and results are normalized to said group on the right graph by a red dashed line when compared by fold change. (**C**) Percentage of 7AAD-positive cells corresponding to each experimental condition on left graph, *n* = 4. Lentivirus yield from two rounds of large-scale packaging on right graph, *n* = 4. (**D**) CXCL13 and CD40L protein concentration in cell supernatant determined by ELISA, *n* = 2. Protein concentration fold changes relative to the virus with EFS promoter. (**E**) Vector maps illustrating 1st Gen EFS control and 2nd Gen LVV that were selected for use in future in vitro and in vivo experiments. Star annotations highlight modifications to vector construct, such as EF1α promoter from EFS, new transgene orientation with CD40L in 1st ORF, CD93 in 2nd ORF, and CXCL13 in 3rd ORF, oPRE operator from WPRE, and kanamycin antibiotic resistance from ampicillin. “(ns)” stands for nonsynonymous describing the type of gene variant.

To enhance the efficacy of our Engineered DC treatment, an optimization study was conducted using Jurkat T-cells. The goal of this study was to increase CD93, CD40L, and CXCL13 transgene expression, modify antibiotic resistance, and optimize manufacturing in lentiviral packaging by modifying transduction parameters, transgene orientation, Multiplicity of Infection (MOI), promoters, and post-transcriptional regulatory elements. To assess the feasibility of the study, the original vector construct (EFS_CD93_CD40L_CXCL13_WPRE) was compared with another vector with a stronger promoter, EF1α, but same gene orientation by evaluating the difference in mean fluorescence intensity (MFI) of CD93 and CD40L at different MOIs of 5, 10, and 20 (Figure 1A). The EF1α virus demonstrated a significantly higher expression level of both CD93 and CD40L. The MFI of CD93 and CD40L in EF1α lentivirus transduced cells was approximately 3.5- and 5.5-fold higher than their expression by the EFS promoter-containing virus across all conditions. A clear correlation between the MOI and the MFI of these genes was observed. The observations above suggest substantially higher CD93 and CD40L expression levels under the EF1α promoter than the EFS promoter in Jurkat T-cells. Based on these results, subsequent experiments were carried out by infecting cells with LVV at an MOI of 5, which was the condition that could best differentiate between the LVVs.

Next, the seven vectors, including the original EFS vector control and the six EF1α vectors with all combinations of transgene orientations, were compared head-to-head by assessing MFI, cytotoxicity, protein secretion, and titer. CD93 expression under the EF1α promoter with CD93 as the 1st ORF demonstrated markedly elevated CD93 expression levels, with a 4- to 6-fold increase in MFI compared to the one with the EFS promoter (Figure 1B). Concurrently, these LVVs also exhibited 2- to 4-fold elevated CD40L expression. The elevated CD93 and CD40L expression under the EF1α promoter aligned with its 5.5- and 4.5-fold increase in expression compared to the EFS promoter from preliminary tests. Among the other four viruses, where CD93 is placed as the 2nd or 3rd ORF, CD93 expression levels were comparable or slightly reduced under the EF1α promoter versus the EFS promoter LVV. LVV with EF1α promoter and CD93 as the 1st ORF, which showed substantial CD93 overexpression, also exhibited a higher percentage of 7AAD^+^ cells (Figure 1C), suggesting higher cell death of transgene-expressing cells. However, no significant increase in 7AAD^+^ cells was observed in the other 4 LVVs with the EF1α promoter. These observations suggest potential cytotoxicity by overexpressing CD93 and highlight the potential correlation between gene orientation and expression.

Based on flow cytometry analysis, the LVVs with CD40L positioning as the 1st ORF and an EF1α promoter had a significant 23- and 6-fold increase in CD40L expression when compared to the LVV with EFS promoter (Figure 1B). When placed in the 2nd position, CD40L expression was 3–4-fold higher in cells transduced with LVV containing EF1α promoter versus the LVV with EFS promoter. Furthermore, at the 3rd position or the 3rd ORF under the EF1α promoter, CD40L expression was 1–2-fold higher compared to the EFS promoter. ELISA measurements of the secreted CD40L protein corroborated the flow cytometry results (Figure 1D). Overall, CD40L expression levels are well-correlated with its position, so that transgenes in the 1st ORF are expressed more than the 2nd or 3rd.

Elevated CXCL13 secretion was observed in all combinations under the EF1α promoter compared to its expression in the LVV containing EFS promoter (CXCL13 secretion was increased by 2.5- to 58.0-fold). The vectors containing CXCL13 as the 1st or 2nd ORF exhibited higher and similar secretion (38.2- to 58.0-fold) among the experimental vectors.

Most LVVs containing the EF1α promoter had about 50% titer yields compared to the LVVs with the EFS promoter, except for one vector. The reduced titer yield observed with LVVs where CD93 was cloned to be the 1st ORF under the EF1α promoter could potentially be attributed to cytotoxicity effects by the overexpression of CD93 (Figure 1C) and the overexpression of CXCL13. High titer yield will offer production advantages for future clinical development, therefore LVV with EF1α promoter containing the gene order as CD40L in the 1st ORF, CD93 in the 2nd, and CXCL13 in the 3rd ORF was selected as the 2nd Gen vector construct for the remaining experimental studies going forward even through it did not had the highest expression increases in the target genes (Figure 1E).

### 3.2. Engineered DCs with 2nd Gen LVV and T-Cell Activation

**Figure 2:**
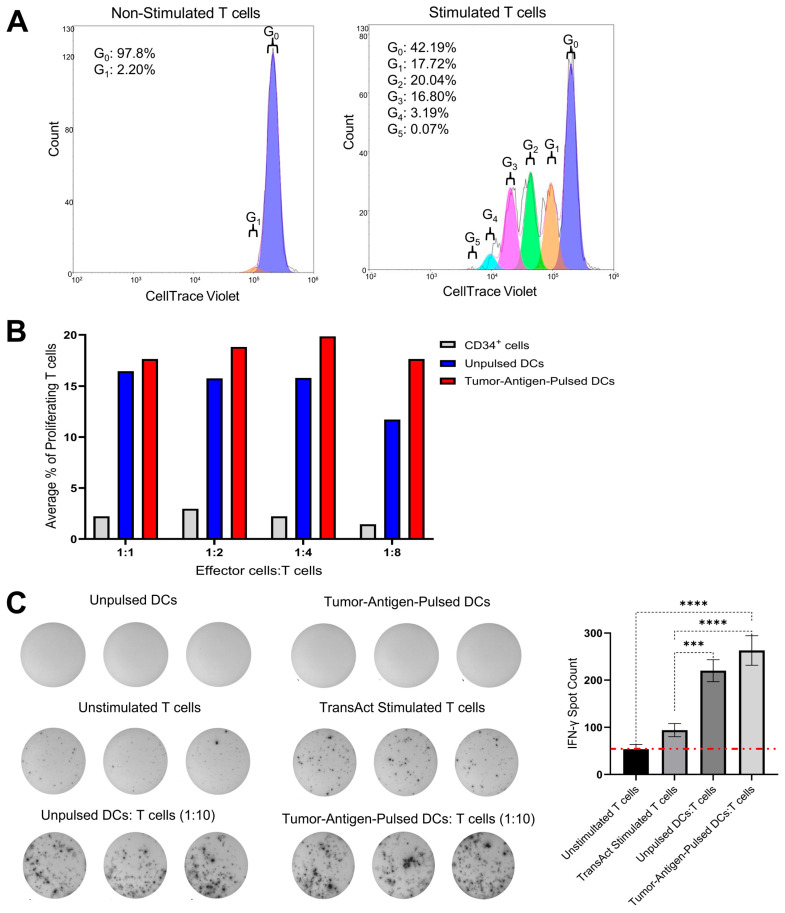
Evaluation of T-cell activation by engineered DCs. (**A**) Flow cytometry analysis of a validation run of the assay using CellTrace Violet staining demonstrating that T cells stimulated with positive control TransAct proliferated, whereas negative control unstimulated T cells showed little proliferation. Flow cytometry results were expressed as the average percentage of total proliferated T cells, *n* = 5 (**B**) MLR assay results, utilizing CellTrace Violet to assess proliferation and T-cell activation, showed that 2nd Gen Engineered DCs exhibited a modest increase in proliferation compared to those co-cultured with control CD34^+^ HSCs, whether they were pulsed with tumor-associated antigens or not. Flow cytometry results were expressed as the average percentage of total proliferated T cells, *n* = 5. (**C**) IFN-γ SFCs per well in different culture conditions, right column graph showing the comparison of SFCs (IFN-γ spot counts) in various co-culture conditions. DCs and T cells were co-cultured at 1:10 ratio (DCs: T cells), *n* = 3. Significance ****—*p* value < 0.0001 and ***—*p* value < 0.001. The red dashed line indicated the threshold of relevant SFC data points in relation to the unstimulated T cell control.

To assess T-cell activation and proliferation in response to co-culture with our allogeneic engineered DCs, a one-way MLR was performed. T-cell stimulation was assessed by flow cytometric analysis following CellTrace Violet labeling, with proliferation reported as the average percentage of dividing T cells. The gating strategy illustrated in Figure S1 was applied to analyze the MLR data. To validate that the assay was working properly on T cells alone, a positive control T-cell activation and proliferation group was established using TransAct (T-cell activator), which induced proliferation across multiple generations (1-G_0_), while 97.8% of unstimulated T cells showed no proliferation (G_0_), *n* = 5 (Figure 2A and Table S1 (raw data)). Next, T-cell proliferation was assessed in co-cultures with effector cells, CD34^+^ HSCs, unpulsed DCs, or tumor-antigen-pulsed DCs at Effector cell/T cell ratios of 1:1, 1:2, 1:4, and 1:8. Both unpulsed and tumor-antigen-pulsed DCs were edited using the 2nd Gen LVV. As shown in Figure 2B, both unpulsed and pulsed DC conditions resulted in higher T-cell activation and a modest increase in the average total percentage of proliferated T cells compared to the CD34^+^ HSC co-culture condition, at all the ratios tested, *n* = 5. Additionally, it was observed that tumor-antigen-pulsed DCs induced greater proliferation than unpulsed DCs at lower DC:T cell ratios, which indicated that T cells stimulated by tumor antigen contributed to a greater percentage of total T-cell activation at lower DC:T cell ratios. This is conferred by the raw data shown in Table S2.

An ELISpot assay was performed to quantify T-cell activation by measuring the IFN-γ secretion following stimulation with 2nd Gen Engineered DCs transduced with a 2nd Gen vector, either unpulsed or pulsed with antigens. T cells were co-cultured with DCs at a 1:10 DC:T cell ratio. Results were expressed as spot-forming cells (SFCs). As shown in Figure 2C, the tumor-antigen-pulsed 2nd Gen Engineered DCs coculture condition resulted in an average of 263 SFCs per well, compared to 220 SFCs per well in the unpulsed DC co-culture condition. Although this difference was not statistically significant, the fact that the 2nd Gen Engineered DCs not only led to T-cell proliferation but also led to higher IFN-γ release encouraged us to test their therapeutic efficacy using humanized mouse models. The pulsed DC condition showed higher T-cell activation compared to the unpulsed condition.

### 3.3. 2nd Gen Engineered DCs Retained Tumor-Inhibitory Function of 1st Gen Engineered DCs Following LVV Modification and Led to Inhibition of Metastases

**Figure 3:**
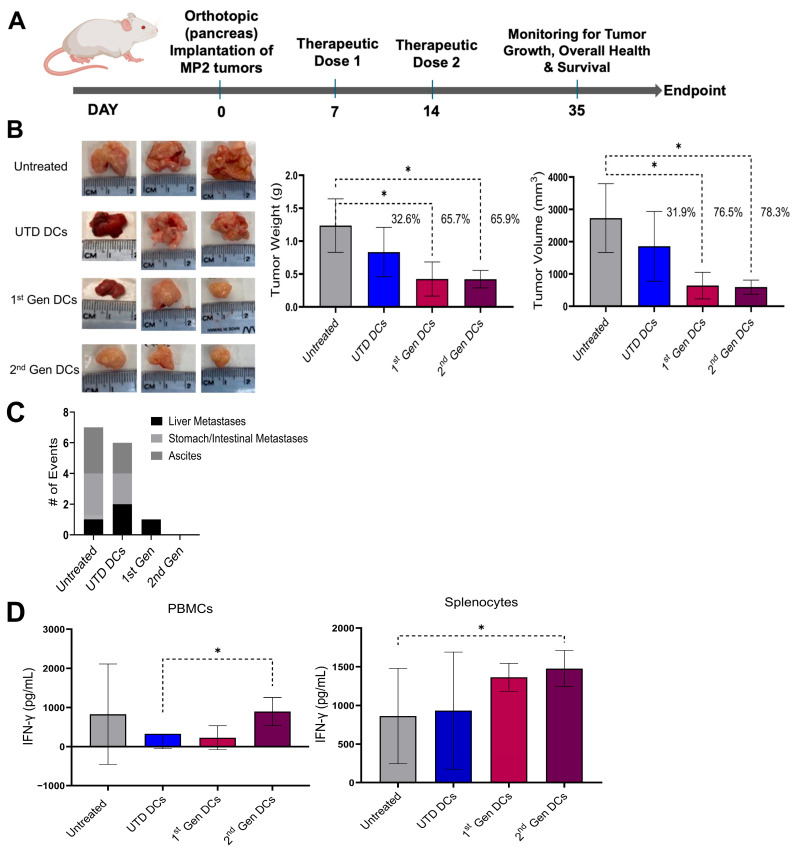
Primary and metastatic tumor remission initiated by treatment with engineered DC in Hu-BLT mouse model. (**A**) Timeline schematic illustrating treatment plan for orthotopic pancreatic tumor-bearing mice. (**B**) Micrographs representing extracted tumors from mice, left; Bar graph illustrating average weight and volume of collected tumors, right. Results are reported as mean ± standard deviation (SD) of tumor weight in grams and tumor volume in mm^3^ (*n* = 3 mice/treatment group). Significance *—*p* value < 0.01–0.05. (**C**) Graph of the number of metastatic lesions in various treatment conditions (*n* = 3 mice/treatment group). (**D**) IFN-γ secretion by PBMCs (right) and Splenocytes (left), isolated from the various treatment groups (mice) and upon treatment with anti-CD3/28 antibodies. Data are presented as mean ± SD.

An in vivo study utilizing an orthotopic pancreatic tumor-bearing Hu-BLT mouse model was performed to compare the 2nd Gen Engineered DCs to the 1st Gen DCs. Aseptic surgery was implemented to orthotopically engraft Hu-BLT mice with 10^6^ human MP2 cells into the pancreas (Figure 3A). Mice were organized into four groups randomly: mice receiving 10^6^ 1st Gen Engineered DCs (pulsed with MP2 lysate), mice receiving 10^6^ 2nd Gen MP2-pulsed Engineered DCs, mice receiving 10^6^ UTD control DCs, and untreated tumor-bearing mice, which served as a negative control. Following the tumor-implantation, DCs were i.d. injected close to the lymph node near the tumor implantation site on D7 and on D14 post-tumor implantation. The tumor growth and health of the mice were monitored until D35, allowing us to capture the details such as immune activation, tumor growth kinetics, and overall survival using humanized mouse models. Post D35 timepoint, mice were sacrificed and subjected to tumor weight and volume analyses, and the evaluation of therapeutic efficacy in the treatment conditions. Our data demonstrates that treatment with 1st and 2nd Gen Engineered DCs significantly reduced tumor growth, yielding 76.5% and 78.3% reductions in tumor volume and 65.7% and 65.9% reductions in tumor weight, respectively, compared with untreated controls or mice receiving LVV control DCs. (Figure 3B). UTD DC-treated mice showed a 32.6% decrease in tumor weight and a 31.9% decrease in tumor volume, indicating that allogeneic DCs can impact tumor growth without antigen loading.

The significant reduction in the tumor weight and volume of mice treated with tumor-antigen pulsed 1st Gen and 2nd Gen Engineered DCs suggests that the expression of transgenes (CD40L, CD93, and CXCL13) combined with antigen pulsing led to elevated immune activation, subsequently leading to therapeutic efficacy. Furthermore, the data showed that the orientation of the transgenes (whether CD40L, CD93, and CXCL13 or CD93, CD40L, and CXCL13) and the upstream promoter (EF1α or EFS) led to similar therapeutic efficacy. Upon analyzing the tumor metastatic lesions, 2nd Gen Engineered DC treatment mice showed the absence of metastatic lesions and ascites compared to the control and UTD DC treatment, and 1st Gen Engineered DC treatment conditions. The metastatic event analyses revealed that in both untreated control and UTD DC treatment conditions, the pancreatic tumors appeared to metastasize to the liver and stomach/intestine. While the 1st Gen DC treatment appeared to inhibit most metastatic events, liver metastases were still observed. In contrast, the optimized 2nd Gen Engineered DCs not only significantly reduced the primary pancreatic tumor but also completely prevented metastatic spread and stromal ascites, while controls bared 77.8% of assessed potential metastasis (Figure 3C).

Next, we isolated and purified PBMCs and splenocytes from mice treated with the 1st and 2nd Gen Engineered DCs compared to control DCs and evaluated the IFN-γ secretion. For these experiments, the PBMCs and splenocytes were further stimulated/activated ex vivo with anti-CD3/28 antibody treatment and evaluated for IFN-γ release. Treatment with 2nd Gen Engineered DC resulted in higher IFN-γ release in PBMC upon stimulation with anti-CD3/28 antibody compared to the 1st Gen DC treatment condition and other control conditions (Figure 3D).

### 3.4. Therapeutic Efficacy of Engineered DCs in Luciferase-Labeled Pancreatic Tumor Model

**Figure 4:**
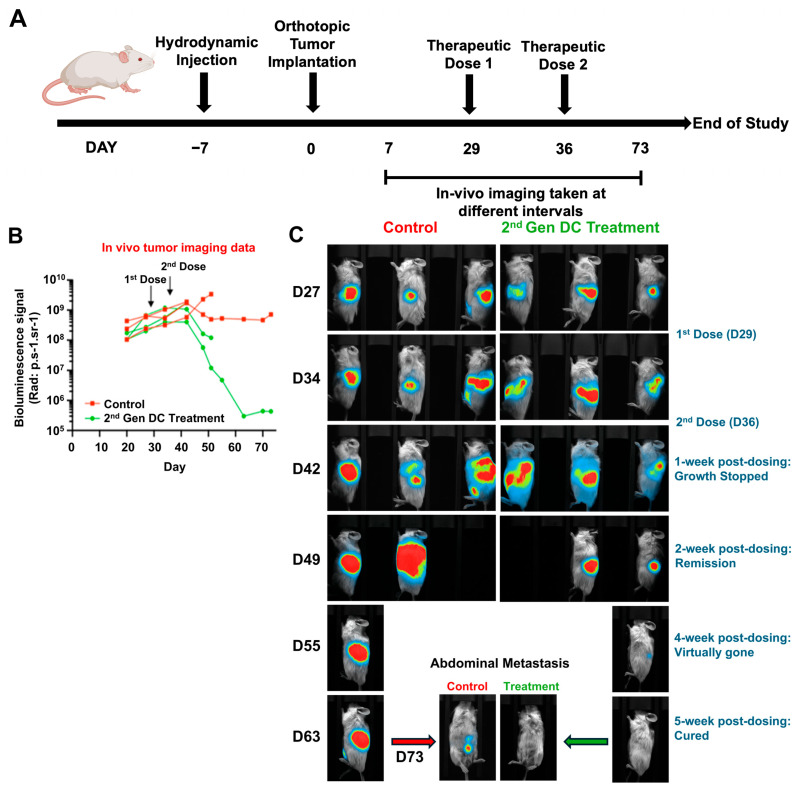
Complete remission of pancreatic tumors and inhibition of metastatic lesions by 2nd Gen Engineered DC treatment in MP2-Luc tumor-bearing humanized mouse model. (**A**) Schematic of the study using orthotopic MP2-Luc tumor-bearing mice. *n* = 3 mice/group (**B**) Representative graph showing tumor growth kinetics, based on the luminescence intensity. (**C**) Images of control and 2nd Gen treatment condition mice using in vivo imaging system. Live imaging system was utilized to capture the tumor growth at various timepoints. Luminescence intensity is identified by the hot spots and decreases in intensity with red being the greatest, followed by green, and then lowest intensity in blue.

Based on our preliminary comparison of 1st and 2nd Gen Engineered DCs in orthotopic pancreatic tumor-bearing mice, and the observed ability of the 2nd Gen DCs to inhibit metastatic progression in humanized mice, we conducted additional studies using MP2-Luc tumor cells (Figure 4A). This approach enabled real-time tracking of primary tumors and metastatic lesions in live mice following treatment with 2nd Gen Engineered DCs. For this study MP2-Luc tumor cells were orthotopically implanted into the pancreas post hydrodynamic injection. Mice were subjected to live in vivo imaging for MP2-Luc tumor growth and tumor metastatic spread (based on the luciferin signal). The tumor growth was calculated based on the intensity of the luciferin signal. Based on the intensity of the luciferin on D27, mice were assigned to either the control group or treatment group (Figure 4B). For this study the tumors were left to grow until D29 (post implantation), and the treatment with 2nd Gen Engineered DCs was initiated. This was performed to assess the therapeutic efficacy of the 2nd Gen Engineered DCs in the late-stage tumor model and upon metastasis of the disease to mimic a clinical patient setup. Mice were given two doses of 2nd Gen Engineered DCs (106/mouse/dose) pulsed with MP2-Luc cell lysates, one on D29 and the second one on D36. Mice were monitored for tumor growth and metastasis post treatment by performing in vivo imaging at frequent intervals (between D34 and D73). In the case of (2nd Gen) Engineered DC treatment condition, tumor growth appeared to be halted after 1 week post-second dose (D42 imaging). From D49, the tumors in the treatment condition showed reduced signal, indicating the tumor was shrinking, a sign of tumor remission. During this period the MP2-Luc tumors in the untreated control groups continued growing as indicated by the increased signal, and one of the three mice in the control group reached endpoint on D49 and another one on D55 (Figure 4B,C, panels D49 and D55). Similarly, in the treatment group, two of the three mice reached endpoint based on treatment-unrelated events (Figure 4C, panel D55). Even though mice in the treatment group reached endpoint, the luciferase images showed a trend of decreased signals where tumors were shrinking.

By D55 onwards, only one mouse in each group was alive, and the study continued to monitor the tumor growth and the presence of metastatic lesions. The in vivo imaging data showed that in the treatment condition, the intensity of the luciferin was minimal, whereas in the control mice, the intensity of the luciferin continued to grow to a saturation point (Figure 4, panel D55). In vivo imaging on D63 and D73 revealed the absence of luciferin in the mouse that received 2nd Gen Engineered DCs, suggesting complete elimination of MP2-Luc cells (Figure 4B). Whereas in the control mouse, in addition to the primary tumor, the appearance of secondary metastatic lesions was observed (Figure 4, D63 and D73), suggesting that the tumor spread across the body. In the 2nd Gen Engineered DC treatment mouse, elimination of the primary tumor and metastatic lesions was observed, suggesting complete remission.

## 4. Discussion

In the present study, we optimized the lentiviral construct by altering its properties like promoters, post-transcriptional regulatory element, transgene orientation, and antibiotic resistance to evaluate the therapeutic potential of 1st and 2nd Gen Engineered DCs in orthotopic humanized pancreatic tumor models, including early- and late-stage disease settings. The 2nd Gen vector construct was determined by evaluating the varying expression of the transgenes and potential cytotoxic effects while also considering lentiviral titer and protein secretion (Figure 1). Ultimately, the vector with CD40L in the 1st ORF, CD93 in the 2nd ORF, and CXCL13 in the 3rd ORF proved to increase transgene expression more than the original construct without sacrificing lentiviral titer and cell viability. Furthermore, protein secretion of CD40L and CXCL13 was greater compared to the 1st Gen vector. After selecting the new vector, its therapeutic efficacy was evaluated in vitro and then compared head-to-head in humanized mouse models.

To evaluate the immunostimulatory potential of our Engineered DCs carrying the 2nd Gen vector, we conducted MLR and ELISpot assays (Figure 2). In the MLR assay, T cells co-cultured with Engineered DCs at decreasing DC:T cell ratios exhibited a dose-dependent increase in cell proliferation, indicating one individual DC was capable of greater T-cell activation. These findings suggest that our Engineered DCs effectively stimulate T-cell responses in a ratio-dependent manner, indicating and confirming previously published data that a single DC can engage with and activate as many as 10 T cells simultaneously. This interaction is crucial for initiating and modulating immune responses for a proper anti-tumor immunity [28,29]. At the lowest DC:T cell ratio (1:8), the difference in total proliferation T cells in tumor-antigen-pulsed Engineered DCs relative to unpulsed Engineered DCs began to exhibit larger differences, suggesting the need for exploration in lower ratios. Future studies will explore even lower DC:T cell ratios to further characterize the proliferative response and determine the optimal conditions for T-cell activation. Although this limitation suggests this observational data should be taken lightly, these results further exemplify DC’s capacity to stimulate multiple cells. Similarly, the ELISpot assay revealed that tumor-antigen-pulsed Engineered DCs induced more IFN-γ-secreting T cells compared to unpulsed DCs. T-cell IFN-γ-secretion was observed in the unpulsed Engineered DC co-culture, which may be caused by an allogeneic response. The tumor-antigen-pulsed optimized DCs led to higher IFN-γ-secretion, suggesting that the antigen-specific response combined with the immunomodulating effects of the three transgenes [30]. Although the difference between pulsed and unpulsed DCs was not statistically significant, this trend underscores the robust T-cell-stimulatory capacity of our tumor-antigen-pulsed DCs transduced with the 2nd Gen vector. These results collectively highlight the potential of our Engineered DCs as potent inducers of T cell responses, warranting further investigation into their application in animal models.

MP2 lysate-pulsed 1st and 2nd Gen DCs significantly reduced tumor burden compared to untreated or UTD DC-treated mice, highlighting the capacity of antigen-loaded DCs engineered to overexpress CD93, CD40L, and CXCL13 to drive robust anti-tumor immunity in the Hu-BLT model. Across three independent experiments, the 1st Gen and 2nd Gen Engineered DCs achieved comparable suppression of primary tumor growth (76.5% and 78.3% reduction in volume, respectively) and tumor mass (65.7% and 65.9% reduction in weight, respectively). The partial anti-tumor effect seen with UTD DCs (~32.6% tumor volume reduction) suggests that allogeneic DCs may exert baseline immune-stimulatory effects independent of transgene expression (Figure 3B). The expression of three transgenes in the Engineered DCs leads to significant tumor reduction in both generations of Engineered DCs, showcasing the importance and role of the immune-modulating properties of CD40L, CD93, and CXCL13.

In our preliminary assessment, a key distinction emerged in the context of metastatic disease control. While the 1st Gen Engineered DCs inhibited most metastatic events, liver metastases remained detectable in some animals. In contrast, the optimized 2nd Gen Engineered DCs not only suppressed primary pancreatic tumor growth but also completely prevented metastatic spread to the liver, stomach, and intestine (Figure 3D). This superior anti-metastatic activity is likely attributable to the combinatorial expression of CD40L, CD93, and CXCL13 and the promoter optimization of the LVV, which may enhance antigen presentation, T cell recruitment, and improved immune modulation in the tumor microenvironment. This is supported by the observation that 2nd Gen Engineered DC treatment induced higher IFN-γ secretion from PBMCs upon stimulation, indicating more potent systemic immune activation.

We further extended these findings to a late-stage disease setting using MP2-Luc tumor cells to enable real-time, non-invasive monitoring of tumor growth and metastatic spread (Figure 4A–B). In this model, treatment with 2nd Gen Engineered DCs initiated on D29 after tumor implantation suppressed tumor progression within one week of the second dose (D36), followed by a trend toward tumor regression (Figure 4B–C). Remarkably, by D63–D73, the single surviving mouse in the treatment arm exhibited complete elimination of luciferase signal, indicating eradication of both primary and metastatic lesions, whereas control mice showed progressive disease with widespread metastases. These results demonstrate that 2nd Gen Engineered DC therapy retains efficacy even against established advanced-stage tumors and may induce durable tumor clearance. These preliminary observational results are highly encouraging and report the effectiveness of the gene-modified allogeneic DCs in treating pancreatic tumors, one of the devastating cancer types.

Our findings are consistent with the results of an already commercially available allogeneic DC vaccine (DCP-001) for acute myeloid leukemia (AML) treatment [31]. In the clinical trial, allogeneic DCs directly presented tumor antigens on allogeneic MHC class I and II to recipient T cells, leveraging the alloimmune response as a natural adjuvant to enhance CD8^+^ and CD4^+^ T cell activation. Additionally, upon rapid clearance by the recipient’s immune system, dying allogeneic DCs release antigens that were taken up by host APCs for indirect presentation on matched MHC molecules, with cross-presentation by host DCs further driving cytotoxic T cell responses against tumor cells. The MHC mismatch itself promoted a pro-inflammatory microenvironment, amplifying T cell priming via cytokines such as IFN-γ and co-stimulatory signals (CD80/CD86), resulting in robust anti-tumor immunity, as evidenced by 70% of patients in NCT03697707 showing WT1-specific T cell responses and 45% achieving MRD-negative status, with minimal toxicities like GVHD.

LVV is commonly used as a gene delivery approach in immunotherapies such as CAR-T products. CAR-T is revolutionary in treating cancers; however, it faces limitations, including variable patient responses, immune-related adverse events like colitis or pneumonitis, and high costs limiting accessibility. LVVs are effective as a gene delivery cargo for ex vivo gene therapy but are hindered by risks of insertional mutagenesis. Our DC vaccine can address the above safety and cost issues. The allogeneic DC is capable of being manufactured at a scalable and significantly lower cost per patient. DC vaccines have been demonstrated to be safe by previous clinical studies, and the allogeneic nature will ensure the LVV gene-edited DCs have a short life span in vivo [17,32,33].

Although encouraging, these findings should be interpreted with caution. The Hu-BLT mouse model provides a partially humanized immune system but does not fully replicate the complexity, heterogeneity, and immune suppression observed in human PC [34]. Additionally, the small cohort sizes, particularly in the late-stage treatment study, limit statistical power and the ability to capture rare outcomes or treatment-associated variability. Additional studies with larger cohorts of mice are needed to fully evaluate the effectiveness of the 2nd Gen Engineered DCs. In this brief report, we are presenting the preliminary observational data that the absence of metastases in the 2nd Gen group is striking; however, the molecular and cellular mechanisms driving this effect remain to be fully elucidated. Additional studies with larger cohorts of mice and immune cell analyses at multiple different timepoints are needed to completely understand the phenomenon.

These studies include validation of current findings in larger and more genetically diverse tumor models, including patient-derived xenografts, coupled with immune cell profiling to identify effector cell subsets, cytokine signaling, associated with metastatic suppression. Detailed mechanistic studies could provide insights into the individual/relative contribution of each transgene (CD40L, CD93, and CXCL13) and how the optimization of their expression leads to therapeutic efficacy.

## 5. Conclusions

In summary, these preliminary observational results suggest that 2nd Gen Engineered DCs, which are now applicable for clinical use, suppress primary tumor growth and prevent metastatic spread in both early- and late-stage disease models, highlighting their promise for clinical development in PC and other aggressive, treatment-refractory solid tumors. We also confirm that the modifications made to the LVV have not compromised the tumor-killing efficacy of engineered cells overexpressing CD93, CD40L, and CXCL13. However, further validation in larger, controlled studies will be essential to confirm these findings and fully define their translational potential.

## Data Availability

All data pertinent to this study is contained within the article.

## Supplementary Materials

The following supporting information can be downloaded at: https://www.mdpi.com/article/10.3390/vaccines13111131/s1, Table S1. Raw Data for MLR Assay Validation for Figure 2A; Table S2. Raw Data for MLR Assay for Figure 2B; Figure S1. MLR Gating Strategy.

## Abbreviations

The following abbreviations are used in this manuscript:

-L	Ligand
AML	Acute Myeloid Leukemia
APC	Antigen Presenting Cells
APC-Cy7	Allophycocyanin-Cy7
ARC	Animal Research Committee
ATCC	American Type Culture Collection
BLT	Bone–Liver–Thymus
CAR-T	Chimeric Antigen Receptor T-cell therapy
CD	Cluster of Differentiation
CELEAG	Local Ethics Committee for Animal Experimentation
CTL	Cytotoxic T Lymphocytes
CXCL-13	Chemokine (C-X-C motif) ligand-13
D	Day
DMEM	Dulbecco’s Modified Essential Medium
FBS	Fetal Bovine Serum
FITC	Fluorescein isothiocyanate
GMP	Good Manufacturing Practice
Gen	Generation
HSC	Hematopoietic Stem Cells
h	Hours
Hu-	Humanized
IACUC	Institutional Animal Care and Use Committee
i.d.	Intradermal Injection
IFN-γ	Interferon-gamma
IMDM	Iscove’s Modified Dulbecco’s Medium
IRB/REB	Institutional Review Board/Research Ethics Board
LVV	Lentiviral Vector
mAbs	Monoclonal Antibodies
mDC	Mature Dendritic Cells
min	Minute
MLR	Mixed Lymphocyte Reaction
MOI	Multiplicity of Infection
MP2	MiaPaca-2
NCG	NOD-Prkdc^em26Cd52^Il2rg^em26Cd22^/GptCrl immunodeficient
ns	nonsynonymous
PBMC	Peripheral Blood Mononucleated Cells
PC	Pancreatic cancer
SD	Standard Deviation
TME	Tumor Microenvironment
UCLA	University of California, Los Angeles
UTD	Untransduced
VB	Vioblue

## References

[1] American Cancer Society (2025). Cancer Facts & Figures 2025. CA: A Cancer Journal for Clinicians.

[2] MD Anderson Cancer Center (2025). Pancreatic Cancer Treatment.

[3] Mazzoccoli L., Liu B. (2024). Dendritic Cells in Shaping Anti-Tumor T Cell Response. Cancers.

[4] Hato L., Vizcay A., Eguren I., Pérez-Gracia J.L., Rodríguez J., Pérez-Larraya J., Sarobe P., Inogés S., de Cerio A.L.D., Santisteban M. (2024). Dendritic Cells in Cancer Immunology and Immunotherapy. Cancers.

[5] Zanotta S., Galati D., De Filippi R., Pinto A. (2024). Enhancing Dendritic Cell Cancer Vaccination: The Synergy of Immune Checkpoint Inhibitors in Combined Therapies. Int. J. Mol. Sci..

[6] Abbaspour M., Esmaeil N., Rezazadeh M., Minaiyan M., Akbari V. (2025). DCs pulsed with hypochlorous acid-treated tumor cell lysates present antigens efficiently and induce CD8+ T cell activation through cross-presentation. Res. Pharm. Sci..

[7] Yoon H.-S., Park Y.G., Cho D.Y., Kim J.-S., Choi J.G. (2025). Antitumor Activity of Ex Vivo Expanded Tumor Antigen Priming Cytotoxic T Cells Against Malignant Melanoma. Anticancer. Res..

[8] Vázquez-Arreguín K. (2025). Novel synthetic peptide-based vaccine shows promise against prostate cancer. Mol. Ther. Oncol..

[9] Hauber I., Beschorner N., Schrödel S., Chemnitz J., Kröger N., Hauber J., Thirion C. (2018). Improving Lentiviral Transduction of CD34(+) Hematopoietic Stem and Progenitor Cells. Hum. Gene Ther. Methods.

[10] Hartupee C., Nagalo B.M., Chabu C.Y., Tesfay M.Z., Coleman-Barnett J., West J.T., Moaven O. (2024). Pancreatic cancer tumor microenvironment is a major therapeutic barrier and target. Front. Immunol..

[11] Olaoba O.T., Yang M., Adelusi T.I., Maidens T., Kimchi E.T., Staveley-O’carroll K.F., Li G. (2024). Targeted Therapy for Highly Desmoplastic and Immunosuppressive Tumor Microenvironment of Pancreatic Ductal Adenocarcinoma. Cancers.

[12] Huerta-Yepez S., Gonzalez J.D., Sheik N., Beraki S., Kathirvel E., Rodriguez-Frandsen A., Chen P.-C., Sargsyan T., Mahammad S., Dybul M.R. (2025). Therapeutic Efficacy of CD34-Derived Allogeneic Dendritic Cells Engineered to Express CD93, CD40L, and CXCL13 in Humanized Mouse Models of Pancreatic Cancer. Vaccines.

[13] Zhang Z., Zheng M., Ding Q., Liu M. (2022). CD93 Correlates With Immune Infiltration and Impacts Patient Immunotherapy Efficacy: A Pan-Cancer Analysis. Front. Cell Dev. Biol..

[14] Elgueta R., Benson M.J., de Vries V.C., Wasiuk A., Guo Y., Noelle R.J. (2009). Molecular mechanism and function of CD40/CD40L engagement in the immune system. Immunol. Rev..

[15] Gao S.H., Liu S.Z., Wang G.Z., Zhou G.B. (2021). CXCL13 in Cancer and Other Diseases: Biological Functions, Clinical Significance, and Therapeutic Opportunities. Life.

[16] Schambach A., Zychlinski D., Ehrnstroem B., Baum C. (2013). Biosafety features of lentiviral vectors. Hum. Gene Ther..

[17] Milone M.C., O’doherty U. (2018). Clinical use of lentiviral vectors. Leukemia.

[18] Yang S., Karne N.K., Goff S.L., Black M.A., Xu H., Bischof D., Cornetta K., Rosenberg S.A., Morgan R.A., Feldman S.A. (2012). A simple and effective method to generate lentiviral vectors for ex vivo gene delivery to mature human peripheral blood lymphocytes. Hum. Gene Ther. Methods.

[19] European Pharmacopeia (2011). Gene Transfer Medical Products for Human Use.

[20] Vandermeulen G., Marie C., Scherman D., Préat V. (2011). New generation of plasmid backbones devoid of antibiotic resistance marker for gene therapy trials. Mol. Ther..

[21] Schambach A., Mueller D., Galla M., A Verstegen M.M., Wagemaker G., Loew R., Baum C., Bohne J. (2006). Overcoming promoter competition in packaging cells improves production of self-inactivating retroviral vectors. Gene Ther..

[22] Mangelinck A., Dubuisson A., Becht E., Dromaint-Catesson S., Fasquel M., Provost N., Walas D., Darville H., Galizzi J.-P., Lefebvre C. (2024). Characterization of CD4+ and CD8+ T cells responses in the mixed lymphocyte reaction by flow cytometry and single cell RNA sequencing. Front. Immunol..

[23] Kaur K., Kozlowska A.K., Topchyan P., Ko M.W., Ohanian N., Chiang J., Cook J., Maung P.O., Park S.-H., Cacalano N. (2019). Probiotic-Treated Super-Charged NK Cells Efficiently Clear Poorly Differentiated Pancreatic Tumors in Hu-BLT Mice. Cancers.

[24] Mittal S., Kumar S., Gupta P., Singh M., Chaluvally-Raghavan P., Pradeep S. (2024). Protocol for the isolation of tumor cell-derived extracellular vesicles followed by in vivo metastasis assessment in a murine ovarian cancer model. STAR Protoc..

[25] Efthymiou A., Mureanu N., Pemberton R., Tai-MacArthur S., Mastronicola D., Scottà C., Lombardi G., Nicolaides K.H., Shangaris P. (2022). Isolation and freezing of human peripheral blood mononuclear cells from pregnant patients. STAR Protoc..

[26] Grosjean C., Quessada J., Nozais M., Loosveld M., Payet-Bornet D., Mionnet C. (2021). Isolation and enrichment of mouse splenic T cells for ex vivo and in vivo T cell receptor stimulation assays. STAR Protoc..

[27] Aiello N.M., Rhim A.D., Stanger B.Z. (2016). Orthotopic Injection of Pancreatic Cancer Cells. Cold Spring Harb. Protoc..

[28] Chudnovskiy A., Pasqual G., Victora G.D. (2019). Studying interactions between dendritic cells and T cells in vivo. Curr. Opin. Immunol..

[29] Pittet M.J., Di Pilato M., Garris C., Mempel T.R. (2023). Dendritic cells as shepherds of T cell immunity in cancer. Immunity.

[30] Lee J., Sheen J.H., Lim O., Lee Y., Ryu J., Shin D., Kim Y.Y., Kim M. (2020). Abrogation of HLA surface expression using CRISPR/Cas9 genome editing: A step toward universal T cell therapy. Sci. Rep..

[31] van de Loosdrecht A.A., van Wetering S., Santegoets S., Singh S.K., Eeltink C.M., den Hartog Y., Koppes M., Kaspers J., Ossenkoppele G.J., Kruisbeek A.M. (2018). A novel allogeneic off-the-shelf dendritic cell vaccine for post-remission treatment of elderly patients with acute myeloid leukemia. Cancer Immunol. Immunother..

[32] June C.H., Sadelain M. (2018). Chimeric Antigen Receptor Therapy. N. Engl. J. Med..

[33] Ribas A., Wolchok J.D. (2018). Cancer immunotherapy using checkpoint blockade. Science.

[34] Brehm M.A., Shultz L.D., Luban J., Greiner D.L. (2013). Overcoming current limitations in humanized mouse research. J. Infect. Dis..

