# Supplementary material for: Modified Hematopoietic Stem Cell-Derived Dendritic Cell Therapy Retained Tumor-Inhibitory Function and Led to Regression of Primary and Metastatic Pancreatic Tumors in Humanized Mouse Models
Source: Vaccines (Basel). 2025 Nov 2;13(11):1131. doi: 10.3390/vaccines13111131 (PMC12656616; doi:10.3390/vaccines13111131)
Supplement: Supplementary file 1 [file vaccines-13-01131-s001.zip › vaccines-3872432-supplementary.pdf]

SUPPLEMENTARY TABLES

Table S1. Raw Data for MLR Assay Validation for Figure 2A. Flow cytometry raw data for validation run of the MLR assay using CellTrace Violet staining demonstrating that T cells stimulated with positive control TransAct proliferated, whereas negative control unstimulated T cells showed little proliferation. Flow cytometry results were expressed as the average percentage of total proliferated T cells. Data shown as the average of 5 wells tested per condition. Gating strategy is illustrated in Figure S1.

	**T cells Not Stimulated**	**T cells Stimulated with MACS GMP T-Cell TransAct**
% viability	83.272	84.05
% CD3+	93.598	79.45
Frequence G_0 (non- proliferating cells)	98.69	43.16
**Frequence 1-G_0 (proliferating cells)**	**1.31**	**56.84**

Table S2. Raw Data for MLR Assay for Figure 2B. MLR assay results, utilizing CellTrace Violet to assess proliferation and T-cell activation, showed that 2nd Gen Engineered DCs exhibited an increase in proliferation compared to those co-cultured with control $CD34^+$ HSCs whether they were pulsed with Tumor associated antigens or not. Whereas we observe the slight increase in T cell proliferation in the DCs pulsed with tumor associated antigens compared to unpulsed DCs, most notably at lower ratios. Flow cytometry results were expressed as the average percentage of total proliferated T cells. Data shown as the average of 5 wells tested per condition. Gating strategy is illustrated in Figure S1.

	**CD34+ Cells**				**Unpulsed DCs**				**Tumor-Antigen-Pulsed DCs**			
Ratio Effector Cells:T cells	1:1	1:2	1:4	1:8	1:1	1:2	1:4	1:8	1:1	1:2	1:4	1:8
% viability	90.088	89.678	83.786	64.608	84.562	87.818	87.85	87.68	82.34	86.112	89.298	87.042
% CD3+	45.454	58.886	73.888	86.968	76.422	85.806	91.22	93.656	81.728	89.584	92.458	94.072
Frequence G_0 (non-proliferating cells)	97.77	97.03	97.77	98.55	83.54	84.25	84.21	88.28	83.30	81.18	80.15	82.36
**Frequence 1-G_0 (proliferating cells)**	**2.23**	**2.97**	**2.23**	**1.45**	**16.46**	**15.75**	**15.79**	**11.72**	**16.70**	**18.82**	**19.85**	**17.64**

SUPPLEMENTAL FIGURE

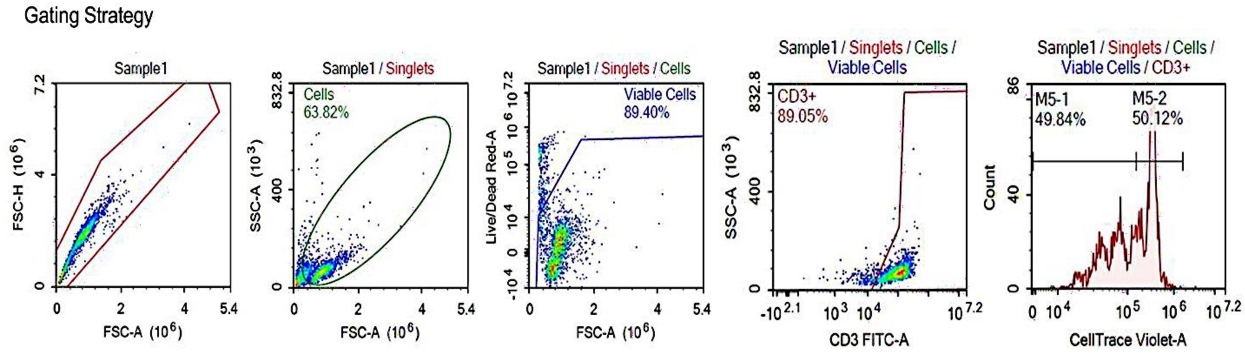

Figure S1. MLR Gating Strategy. The plots above picture the gating strategy implemented for the analysis of the MLR results. First, cells are gated by morphology, the singlets are isolated, followed by viable cells (% viability). Once the isolated viable singlets are selected the CD3+ marker (% CD3+) was used to identify T cells followed by examination of the CellTrace Violet-A, which was used to identify and quantify the level of T cell proliferation and generations of said proliferating T cells.
